# *Bifidobacterium adolescentis* SBT2786 Improves Sleep Quality in Japanese Adults with Relatively High Levels of Stress: A Randomized, Double-Blind, Placebo-Controlled Study

**DOI:** 10.3390/nu16111702

**Published:** 2024-05-30

**Authors:** Hiroki Murakami, Taro Ko, Haruka Ouchi, Toshiharu Namba, Shukuko Ebihara, Shunjiro Kobayashi

**Affiliations:** 1 Milk Science Research Institute, MEGMILK SNOW BRAND Co., Ltd., 1-1-2 Minamidai, Kawagoe, Saitama 350-1165, Japan; hiroki-murakami@meg-snow.com (H.M.); t-koh@meg-snow.com (T.K.); haruka-ouchi@meg-snow.com (H.O.); 2 CPCC Company Limited, 3-3-10 Nihonbashi Hongokucho, Chuo-ku, Tokyo 103-0021, Japan; t.nanba@cpcc.co.jp; 3 Chiyoda Paramedical Care Clinic, 3-3-10 Nihonbashi Hongokucho, Chuo-ku, Tokyo 103-0021, Japan

**Keywords:** *Bifidobacterium adolescentis* SBT2786, clinical study, sleep, stress

## Abstract

Sleep disorders associated with lifestyle changes and unhealthy habits are major public health concerns. Our previous study showed that *Bifidobacterium adolescentis* SBT2786 has a potent sleep-promoting effect on fruit flies. Fruit flies share many similarities with mammals, making them suitable model organisms for studying sleep. Thus, in the present study, we conducted a randomized, double-blind, placebo-controlled clinical trial to test whether SBT2786 has sleep-enhancing effects in humans. In this study, 61 participants in the SBT2786 group and 65 participants in the placebo group were analyzed. The results showed that SBT2786 increased sleep time; however, it predominantly increased light sleep and did not improve subjective sleep quality. Interestingly, mood improvement was observed. A subgroup analysis was conducted on participants with high stress levels, and results showed that these participants experienced an increase in sleep duration and an improvement in sleepiness upon waking up and reported feeling well-rested during the day. We concluded that SBT2786 may improve sleep quality, particularly in individuals experiencing high levels of stress, and that SBT2786 can be used as a dietary supplement to improve sleep and mood.

## 1. Introduction

Sleep disorders and disturbances have become increasingly prevalent and significant public health issues in contemporary society. This increase is largely due to a variety of factors, including drastic lifestyle changes, the prevalence of irregular work schedules, and the adoption of unhealthy habits [1,2]. Sleep deprivation is not only associated with increased daytime sleepiness and decreased productivity. Sleep deprivation is strongly associated with various physical and mental health conditions, including cardiovascular diseases, obesity, diabetes, and depression [3,4,5,6]. These sleep-related problems not only affect individuals but also burden the economy significantly, owing to reduced societal productivity and increased medical costs [1]. Given the prevalence and severity of these problems, the discovery and development of effective and safe strategies for improving sleep quality is a public health challenge.

Recently, the health-promoting potential of intestinal bacteria, including lactic acid bacteria and bifidobacteria, has received increasing attention. Beneficial bacteria are known to have positive effects on host health. They modulate the intestinal flora, boost immune function, and produce bioactive compounds that contribute to overall host health [7,8,9]. Additionally, a growing body of research suggests that the gut microbiota, a community of microorganisms that reside in the intestine, may have a profound influence on the central nervous system. This influence extends to the regulation of various brain functions, including sleep, through a complex communication network known as the gut–brain axis [10,11,12,13,14].

Fruit flies (*Drosophila melanogaster*) exhibit numerous similarities with mammals, particularly in the interaction between the intestinal flora and host, which relates to immune function and metabolism [15]. Furthermore, fruit flies are also known to enter a sleep-like state, and their sleep mechanism is similar to that of mammals, making them a suitable model organism for studying sleep [16,17,18]. In our previous study, we assessed the effects of various human gut and food-associated bacterial species on the sleep behavior of fruit flies by administering them orally. Among the species tested, we found that *Bifidobacterium adolescentis* SBT2786 had the most potent sleep-promoting effect [19]. Therefore, SBT2786 may be effective in improving sleep in humans.

In the present study, we aimed to assess the effect of *B. adolescentis* SBT2786 on sleep quality and evaluate its safety in Japanese adults using a randomized, double-blind, placebo-controlled trial.

## 2. Materials and Methods

### 2.1. Study Design and Ethical Statement

This randomized, double-blind, placebo-controlled, parallel-group trial (RCT) was conducted from February to December 2023 in Japan. The study protocol underwent ethical review and validation by the Ethics Review Committee of Chiyoda Paramedical Care Clinic on 17 February 2023 (approval number: 23021701). It was registered in the University Hospital Medical Information Network Clinical Trials Registry (UMIN ID: UMIN000050446) prior to commencement. The study adhered to the principles of the Declaration of Helsinki and the Ethical Guidelines for Life Sciences and Medical Research Involving Human Subjects, as stipulated by the Ministry of Education, Culture, Sports, Science and Technology; the Ministry of Health, Labor and Welfare; and the Ministry of Economy, Trade and Industry in Japan. The study prioritized the protection of the subjects’ human rights throughout its duration, and written informed consent was obtained from all subjects.

The study design included a screening test, a one-week pre-observation period, and an intervention period. The participants were required to complete a daily questionnaire about their living conditions and schedule three physician visits during the study period (at the time of the screening test, week 0, and week 4).

### 2.2. Participants

Healthy Japanese men and women, aged 30–59 years, with a body mass index (BMI) of <30 kg/m^2^, who were dissatisfied with their sleep quality, employed on weekdays, and had the habit of retiring to bed by 24:00, were recruited. However, individuals were excluded from participation if they met any of the exclusion criteria listed in Supplementary Table S1.

During the study period, participants were required to maintain their lifestyle habits as before the study, especially sleeping habits, eating habits, and exercise; avoid consuming foods for specified health uses, foods with function claims, health foods (including dietary supplements), etc.; refrain from consuming caffeinated beverages such as coffee, green tea, black tea, oolong tea, and nutritional drinks after 8:00 p.m.; abstain from alcohol, do complete bathing at least 2 h before bedtime; avoid exposure to blue light, such as using a computer or smartphone, for 1 h before bedtime (except when entering the daily logbook); and if they had a habit of exercising at night, refrain from exercising for 2 h before bedtime during the examination period.

### 2.3. Sample Size

For the primary outcome measures, which included either the scores of any sleep variables as measured using an electroencephalogram (EEG) or any scores from the Oguri–Shirakawa–Azumi Sleep Inventory ver. MA (OSA-MA), we hypothesized a moderate difference (d = 0.5) between the group consuming the active food and the one consuming the placebo food. When conducting an independent two-sample *t*-test with a two-sided significance level of 5% and power of 80%, 64 participants were required per group. However, considering an anticipated dropout rate of approximately 10% during the trial period, we set the sample size to 70 participants per group.

### 2.4. Randomization

To minimize bias related to factors such as age, sex, and the Japanese version of the Pittsburgh Sleep Quality Index (PSQI-J), OSA-MA, and EEG measurements, the participants were divided into two groups. Subsequently, each group was assigned to either the SBT2786 or placebo group by an independent controller. The allocation list was sealed by the controller and maintained until the designated unsealing time was reached.

### 2.5. Test Foods

The test foods included active capsules containing *B. adolescentis* SBT2786 and placebo capsules without *B. adolescentis* SBT2786. Active capsules were prepared by mixing and encapsulating *B. adolescentis* SBT2786 powder and starch. Active capsules were formulated to deliver more than 1.0 × 10^11^ cells of *B. adolescentis* SBT2786 when four capsules were consumed. Meanwhile, placebo capsules were prepared using starch as a substitute for *B. adolescentis* SBT2786. The test foods were indistinguishable in terms of color, shape, and taste. Participants were instructed to consume four capsules daily over a period of 4 weeks.

### 2.6. Outcomes

The primary outcome of this study was sleep quality, which was assessed using sleep EEG measurements and OSA-MA [20]. Secondary outcomes included measurements using PSQI-J [21], the Japanese version of the Epworth Sleepiness Scale (ESS) [22], the Profile of Mood States second edition (short version) (POMS2) [23], and the visual analog scale (VAS) for sleep or fatigue, as well as levels of salivary amylase and blood growth hormone.

### 2.7. Measurement and Analysis of Sleep Electroencephalogram

Sleep EEG measurements of overnight brain activity were recorded using a single-channel electroencephalograph (Sleep Scope; SleepWell Co., Ltd., Osaka, Japan). Sleep EEG data were recorded for seven consecutive nights during the pre-observation period (before the intervention) and the last week of the intervention period. Three problem-free days were selected for analysis, and their averages were calculated for each sleep variable. If only two problem-free days were available, the average was calculated. If there were one day or less, the participants were excluded from the analysis. Data retrieval and analysis from the raw EEG data were outsourced to SleepWell Co., Ltd., which provided automatic classification that yielded a sleep quality index for each participant.

### 2.8. Sleepiness and Physical Condition Assessment Using Questionnaires

The participants were asked to complete the OSA-MA and VAS (sleep) upon waking each day during the pre-observation period and the last week of the intervention period. The results from the day following each EEG measurement were averaged and used as the data. Participants completed the ESS and VAS (fatigue) after 16:00 during the same period, and the results from the same day as each EEG measurement were averaged and used as the data. The VAS was scored using a 100 mm scale, with 0 mm representing the worst (in sleep) or best (in fatigue) condition and 100 mm representing the best (in sleep) or worst (in fatigue) condition. Participants also completed the PSQI-J and POMS2 during screening, pre-testing, and at the end of the study.

### 2.9. Evaluation of Biological Indicators

Stress levels were assessed by measuring salivary amylase levels. Briefly, saliva samples were collected at baseline (week 0) and after the intervention (week 4). The samples were tested for amylase activity at IMUH Co., Ltd. (Tokyo, Japan). To evaluate sleep status, blood growth hormone levels were measured at the same time points as the saliva samples (weeks 0 and 4). Blood growth hormone levels were measured by BML, Inc. (Tokyo, Japan).

### 2.10. Safety Assessments

To evaluate the safety of *B. adolescentis* SBT2786, a medical interview with a physician, physical measurements (height and weight), and physiological tests (blood pressure and pulse rate) were performed at screening and at weeks 0 and 4. In addition, hematological, blood biochemistry, and general urine tests were performed at screening and at week 4.

Hematological tests included white and red blood cell counts, hemoglobin levels, hematocrit levels, and platelet counts. Blood chemistry tests encompassed total protein, albumin, total bilirubin, alkaline phosphatase, lactate dehydrogenase, aspartate aminotransferase, alanine aminotransferase, γ-glutamyltransferase, creatine kinase, total cholesterol, triglycerides, high-density lipoprotein cholesterol, low-density lipoprotein cholesterol, urea nitrogen, creatinine, uric acid, sodium, potassium, chloride, calcium, glucose, and glycated hemoglobin. A general urine test was performed to assess protein, sugar, urobilinogen, bilirubin, and occult blood levels. All measurements were performed by BML, Inc. (Tokyo, Japan).

Additionally, the participants were instructed to maintain a daily record of their intake of test samples, lifestyle changes (including diet, alcohol consumption, exercise levels, bowel movements, and sleep duration), work status, menstrual cycle (for female participants), use of pharmaceutical drugs, and visits to medical institutions.

### 2.11. Statistical Analysis

Statistical analyses were performed using Microsoft Excel and IBM SPSS Statistics version 26. Data were expressed as mean ± standard deviation. Group comparisons of EEG data, questionnaire scores, and biomarker levels were performed using unpaired Student’s *t*-test, Welch’s *t*-test, or analysis of covariance (ANCOVA) with pre-intake values as covariates. Fisher’s exact test was used to examine participant demographics. A *p*-value of <0.05 indicated a statistically significant difference.

## 3. Results

### 3.1. Participants

**Figure 1:**
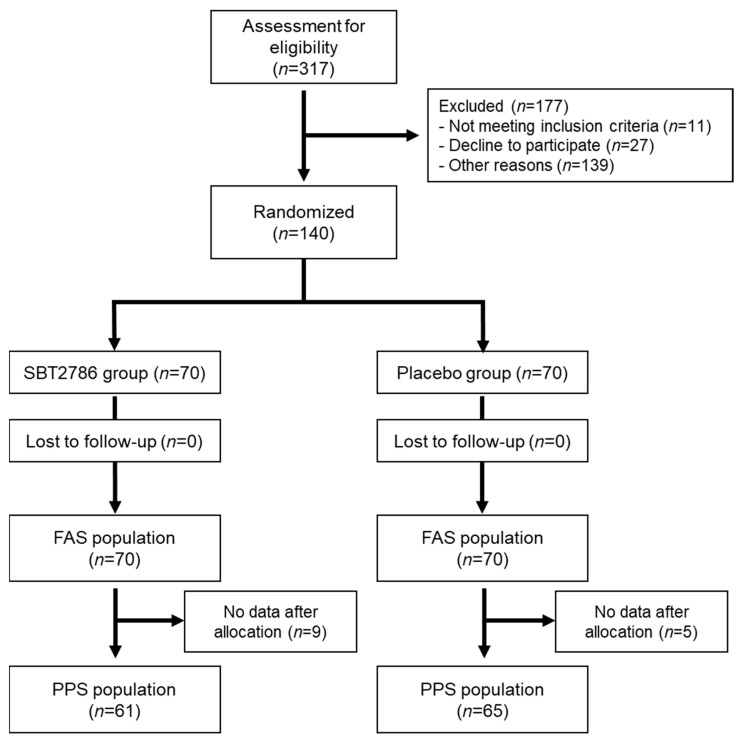
Flow chart of participant recruitment and participation. FAS: full analysis set, PPS: per protocol set.

A flowchart of participation is shown in Figure 1. A screening test was conducted on 317 candidates who provided consent to participate in the trial, wherein 140 participants were selected. These participants were randomly assigned to two groups (SBT2786 and placebo groups, *n* = 70) and consumed the test food. During the consumption period, there were no participant dropouts, the consumption rate of the test food was 100%, and all 140 participants completed the trial. Therefore, these 140 participants constituted the full analysis set (FAS) and were subjected to safety analyses. However, 14 participants (nine from the SBT2786 group and five from the placebo group) were unable to obtain the prescribed number of EEG data (2 days or more) due to noise. The remaining 126 participants (61 from the SBT2786 group and 65 from the placebo group) underwent the per protocol set (PPS) and were analyzed for the primary and secondary outcomes.

### 3.2. Participant Information

Table 1 shows the participant’s background. No significant differences were observed in any background characteristic between the two groups. In addition, hematological and biochemical blood test results were within standard ranges.

### 3.3. Primary Outcome

**Figure 2:**
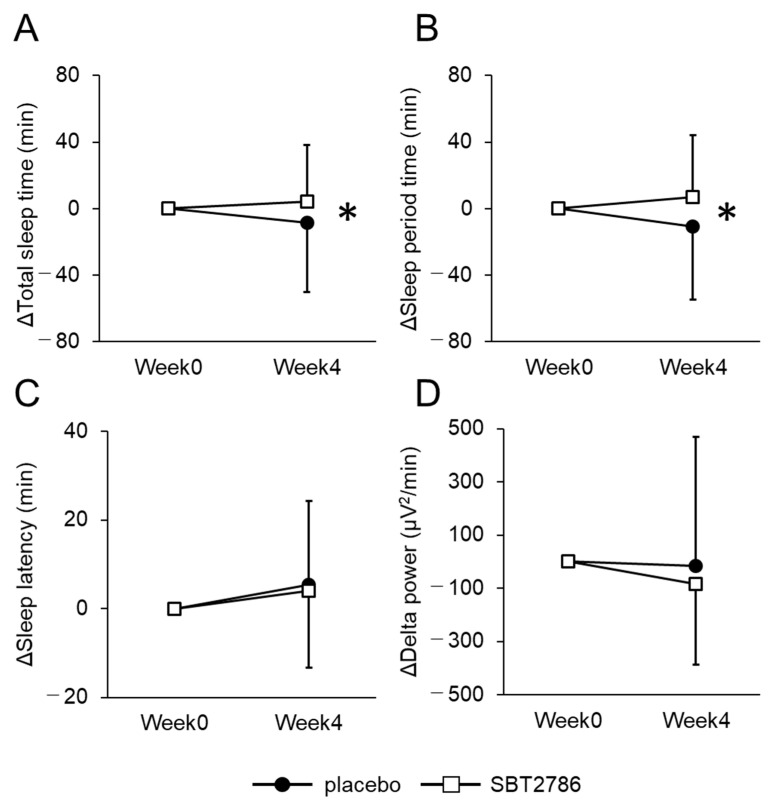
Changes in EEG-derived sleep parameters. (**A**) Total sleep time, (**B**) Sleep period time, (**C**) Sleep latency, (**D**) Delta power in SPT. Values are presented as means ± SD. * *p* < 0.05, significant differences between the SBT2786 and placebo groups (ANCOVA with each initial value as a covariate).

The data derived from the EEG measurements are presented in Figure 2 and Supplementary Table S2. Compared with the placebo group, the SBT2786 group exhibited an elongation in sleep time (total sleep time, sleep period time), along with an increase in REM sleep time and wakefulness. However, no significant differences in the subjective evaluation using the OSA-MA were observed between the SBT2786 and placebo groups (Supplementary Table S3). These results suggest that although SBT2786 facilitated an increase in light sleep, this effect was not subjectively discernible.

As a post hoc analysis, we analyzed whether sex and age affected the sleep-improving effects of SBT2786 using a two-way analysis of variance. The results showed that sex and age had little effect on the sleep-improving effects of SBT2786 (Supplementary Tables S4 and S5).

### 3.4. Secondary Outcomes

**Figure 3:**
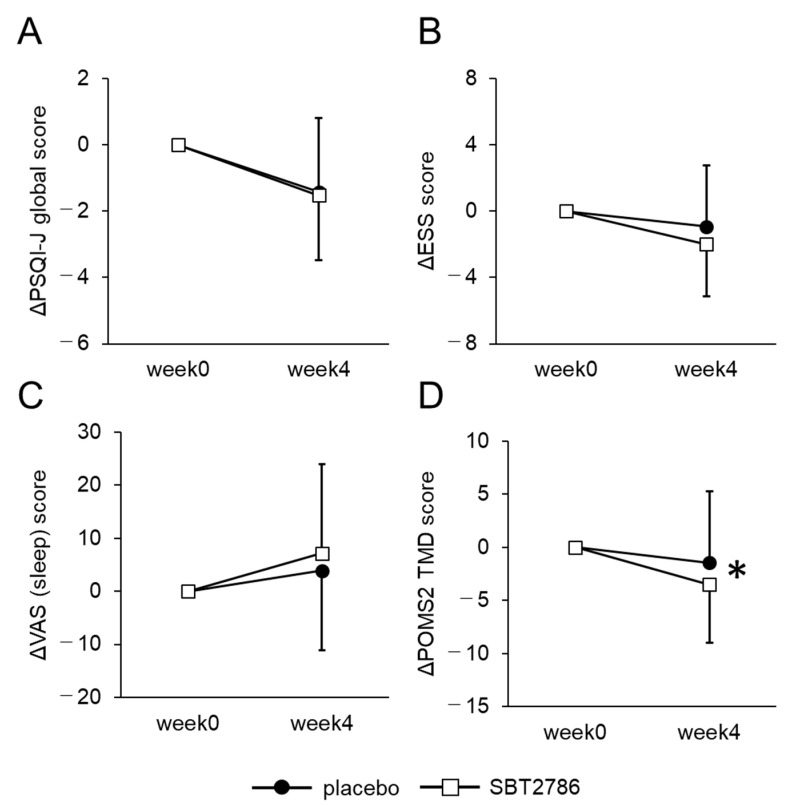
Changes in sleepiness and physical condition assessed using questionnaires. (**A**) PSQI-J global score, (**B**) ESS, (**C**) VAS (sleep), (**D**) POMS2 TMD score. Values are presented as means ± SD. * *p* < 0.05, significant differences between the SBT2786 and placebo groups (ANCOVA with each initial value as a covariate).

Figure 3 and Supplementary Table S6 show the secondary outcomes. No significant differences in the PSQI-J, ESS, or VAS (sleep and fatigue) scores were observed between the SBT2786 and placebo groups. In POMS2, which assessed the mood state of the participants, the Total Mood Disturbance (TMD) score showed a significant improvement in the SBT2786 group. Additionally, no significant variations were found in salivary or blood biomarkers (Supplementary Table S6). These results suggest that while SBT2786 does not have a significant effect on sleep measures, fatigue, or biomarkers, it does have a positive effect on the mood state of the participants.

### 3.5. Subgroup Analysis

Subgroup analysis was performed to elucidate the effect of SBT2786 on stress levels. The close relationship between stress levels and sleep state is well established [24]. Therefore, we used salivary amylase, a marker widely recognized for its reliability in stress measurements [25,26,27], as an indicator of stress level. Participants with pre-intervention salivary amylase activity above the mean were categorized into the high-stress subgroup, and the effects of SBT2786 were analyzed within this group. A total of 55 participants showed above-average salivary amylase activity (26 from the SBT2786 group and 29 from the placebo group). No significant differences were observed in the background information of the participants (Table 2).

**Figure 4:**
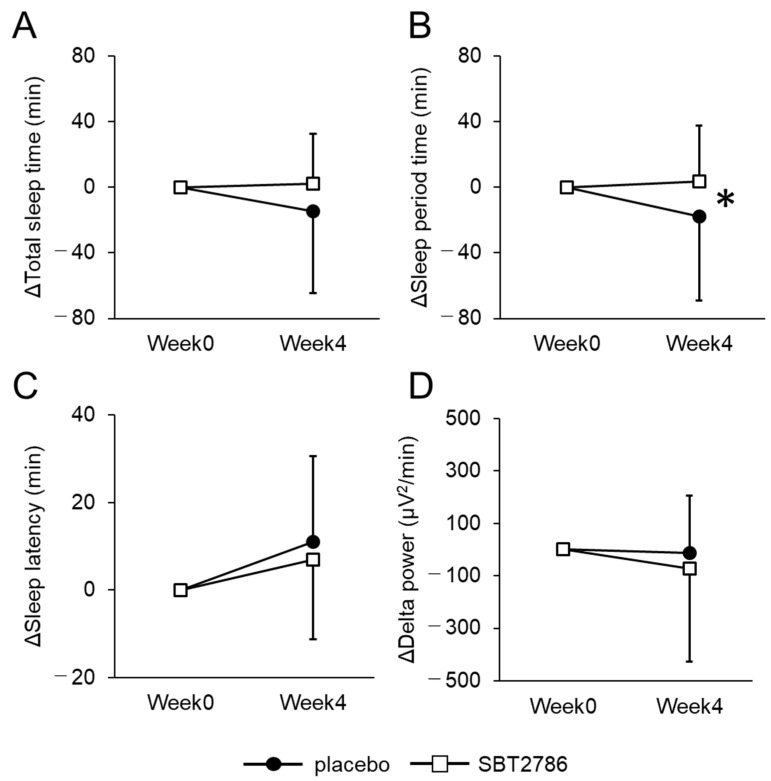
Changes in EEG-derived sleep parameters in the subgroup analysis. (**A**) Total sleep time, (**B**) Sleep period time, (**C**) Sleep latency, (**D**) Delta power in SPT. Values are presented as means ± SD. * *p* < 0.05, significant differences between the SBT2786 and placebo groups (ANCOVA with each initial value as a covariate).

**Figure 5:**
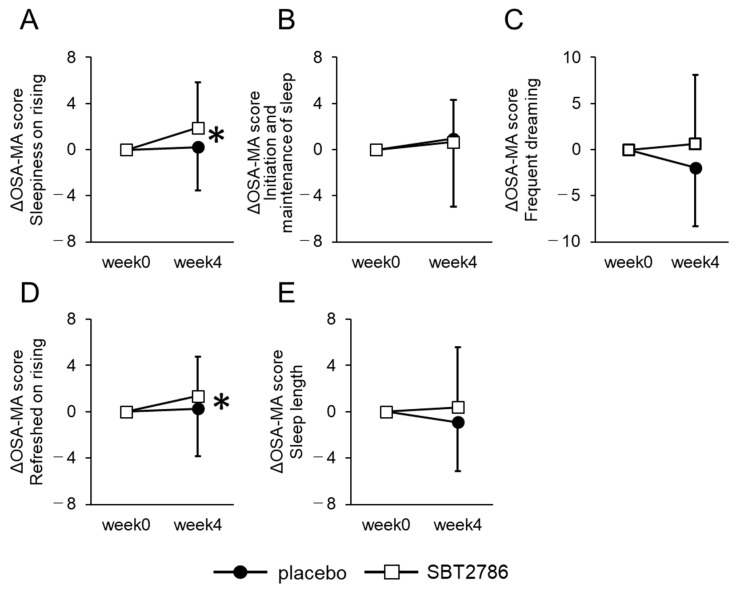
Changes in OSA-MA analysis on sleep quality in the subgroup analysis. (**A**) Sleepiness on rising, (**B**) Initiation and maintenance of sleep, (**C**) Frequent dreaming, (**D**) Refreshed on rising, (**E**) Sleep length. Values are presented as means ± SD. * *p* < 0.05, significant differences between the SBT2786 and placebo groups (ANCOVA with each initial value as a covariate).

**Figure 6:**
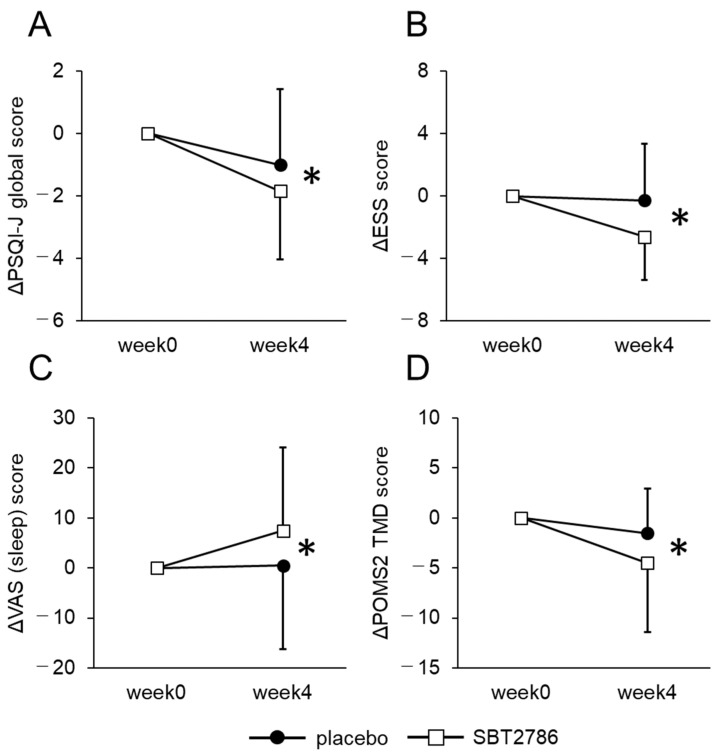
Changes in sleepiness and physical condition in the subgroup analysis. (**A**) PSQI-J global score, (**B**) ESS, (**C**) VAS (sleep), (**D**) POMS2 TMD score. Values are presented as means ± SD. * *p* < 0.05, significant differences between the SBT2786 and placebo groups (ANCOVA with each initial value as a covariate).

From the EEG measurements, we observed an increase in sleep period time and a decrease in wake time in the last 2 h of sleep in the SBT2786 group compared with the placebo group (Figure 4 and Supplementary Table S7). In addition, in the OSA-MA, scores for “Sleepiness on rising” and “Refreshed on rising” improved significantly in the SBT2786 group (Figure 5 and Supplementary Table S8). In terms of secondary outcomes from the sleep questionnaires, the SBT2786 group showed significant improvements in the PSQI-J, ESS, and VAS scores (sleep), along with an improvement in the POMS2 TMD score (Figure 6 and Supplementary Table S9). Conversely, no significant differences were observed in daytime fatigue (VAS (fatigue)) or biological markers.

These findings suggest that the consumption of SBT2786 can enhance sleep quality and alleviate the psychological effects of stress in individuals with high stress levels.

### 3.6. Safety Assessment

In this trial, adverse events were observed in six (seven events) and 12 (17 events) participants in the placebo and SBT2786 groups, respectively. No significant differences in terms of the occurrence of adverse events were observed between the groups. Furthermore, based on the results of interviews and inquiries with the participants, the principal investigator judged that there was no causal relationship between the occurrence of all adverse events and the intake of the test food, and no side effects were observed.

Additionally, when evaluating the changes in clinical test values (Supplementary Table S10), significant differences and temporal changes were observed in some items between the two groups. The principal investigator determined that all pre- and post-intervention clinical test item values were within the range of physiological variation and that the differences and temporal changes between the two groups did not negate safety. Based on these findings, we concluded that there are no safety concerns regarding the consumption of *B. adolescentis* SBT2786 over a four-week period.

## 4. Discussion

This study has significant implications, as it demonstrates the effectiveness of SBT2786 in both *D. melanogaster* and humans. In a previous study, we found that SBT2786 prolongs nighttime sleep duration in *D. melanogaster* [19]. Given that *D. melanogaster* shares a sleep control mechanism with humans [18], we hypothesized that SBT2786 might also be effective in humans. To test this hypothesis, we conducted a double-blind, placebo-controlled trial to investigate whether SBT2786 could enhance the sleep quality of individuals dissatisfied with their sleep. After a 4-week intervention, SBT2786 treatment resulted in extended sleep duration. This represents a successful example of using *D. melanogaster* for screening before efficacy testing in humans. Thus, the utility of *D. melanogaster* as a model organism is expected to increase.

We also observed that the effectiveness of SBT2786 in humans was influenced by psychological conditions, particularly stress levels. Stress is known to have both positive and negative effects on sleep. Short-term stress increases the amount of sleep [28]. This is thought to be because of the need to recover and adapt to the changed environment. On the other hand, chronic stress leads to sleep disturbances, such as reduced deep sleep and disrupted sleep–wake cycles [29,30,31]. This is considered to be one of the detrimental effects of stress on the central nervous system. Salivary amylase is recognized as an indicator of both acute and chronic stress states [25,32]. In this study, we used salivary amylase to assess the stress levels of participants, focusing particularly on those with high stress levels, which may also have disturbed sleep. Our findings revealed that the consumption of SBT2786 led to an increase in sleep duration, as observed using EEG, an improvement in sleepiness upon waking and during the day, the feeling of being well-rested, and a reduction in fatigue upon waking, as assessed by the questionnaires. Additionally, the PSQI-J scores were below the cutoff of 5.5. These results suggested that SBT2786 not only prolongs the duration of sleep but also enhances sleep quality in individuals with high stress levels. In recent years, sleep restfulness, the feeling of restfulness experienced upon waking in the morning, has been suggested to be more important than the length of sleep for maintaining health in adults [33]. Epidemiological studies have suggested that sleep restfulness is associated with the risk of myocardial infarction, angina, stroke, heart failure, and atrial fibrillation [34,35]. As SBT2786 improved sleepiness and fatigue upon waking up, it may have contributed to increased sleep restfulness. In addition, the TMD scores on the POMS2 test improved with SBT2786 consumption. The POMS2 is a psychological assessment tool used to assess individual mood states and create an overall mood profile [23]. These results suggest that SBT2786 alleviates the psychological effects of stress and potentially improves sleep quality.

*B. adolescentis* is a member of the adult human gut microbiota. A growing body of evidence suggests that the gut microbiota significantly influences brain function. For example, lactic acid bacteria have been demonstrated to improve sleep conditions by modulating the vagus nervous system [36,37]. Additionally, short-chain fatty acids produced by the gut microbiota can stimulate the hypothalamic–pituitary–adrenal axis and affect central neurotransmission via the mucosal immune system [38,39]. Moreover, the metabolism of non-digestible fibers by the gut microbiota provides neurotransmitters, including gamma-aminobutyric acid (GABA), which is the primary inhibitory neurotransmitter in the central nervous system [40]. *B. adolescentis* has been identified as a key member of the human gut microbiota in the production of GABA [41]. Furthermore, *B. adolescent* has been demonstrated to exert anxiolytic and antidepressant effects by inhibiting the activity of inflammatory cytokines, such as NF-κB [42,43,44]. In light of the aforementioned studies, it is possible that the effects of SBT2786 are mediated by either GABA production or the suppression of inflammatory cytokines. Further research is required to clarify the potential mechanisms underlying the effects of SBT2786 on brain function.

In addition, heat-killed *L. gasseri* CP2305 and *Lactobacillus brevis* SBC8803 have been reported to alter the gut microbiota and improve sleep quality [45,46]. In the present study, we used *B. adolescentis* SBT2786 (1.0 × 10^11^ <); however, the number of live bacteria in the test food consumed was unclear. *Bifidobacterium* spp. are obligate anaerobes, and *B. adolescentis* is particularly oxygen-sensitive and unable to grow even under low-oxygen conditions [47]. This suggests that the number of live bacteria in the test food may have been extremely low. Our previous study reported that even heat-killed SBT2786, as well as unkilled SBT2786, resulted in prolonged sleep duration at night and shortened sleep latency [19]. Based on these results, live SBT2786 cells are not essential for improving sleep quality; instead, they may act as a prebiotic that induces changes in the gut microbiota or as a biogenic substance that directly affects the host.

However, the limited sample size and restriction of the participants to a specific population may have affected the generalizability of the results. Additionally, the short duration of the study and specific time points at which measurements were taken may not have provided a complete picture of the effects of SBT2786. It is noteworthy that the questionnaires used in this study were subjective. Despite these limitations, this study yielded positive results under the best possible conditions.

## 5. Conclusions

In the present study, we conducted a randomized, double-blind, placebo-controlled clinical trial to test whether SBT2786 has sleep-enhancing effects in humans. The results showed that SBT2786 may improve sleep quality, particularly in individuals experiencing high levels of stress. These findings indicate that SBT2786 can be used as a dietary supplement to improve sleep and mood.

## Figures and Tables

**Table 1:** Participant information.

Items	Unit	Placebo			SBT2786			*p*-Value
Number of Subjects		65			61			
Sex	Male/Female	30/35			32/29			0.593
Age	Years	46.7	±	7.3	46.1	±	7.0	0.642
Height	cm	164.1	±	7.1	166.2	±	8.4	0.139
Weight	kg	61.1	±	10.2	62.4	±	11.6	0.516
BMI	kg/cm^2^	22.6	±	2.9	22.4	±	2.8	0.730
Systolic blood pressure	mmHg	119.4	±	13.8	118.7	±	13.2	0.784
Diastolic blood pressure	mmHg	74.6	±	9.9	74.6	±	11.9	0.985
Pulse rate	beat/min	68.9	±	9.5	71.7	±	10.0	0.117
PSQI-J global score		7.6	±	1.5	7.6	±	1.6	0.936
Test sample consumption	%	100.0	±	0.0	100.0	±	0.0	-

Values are shown as mean ± SD. No significant differences were observed between groups (Fisher’s exact test or *t*-test). PSQI-J, Japanese version of the Pittsburgh Sleep Quality Index.

**Table 2:** Background of the participants in the subgroup analysis.

Items	Unit	Placebo			SBT2786			*p*-Value
Number of Subjects		29			26			
Sex	Male/Female	12/17			12/14			0.789
Age	Years	48.4	±	6.6	47.7	±	6.9	0.708
Height	cm	164.3	±	6.5	165.8	±	8.2	0.465
Weight	kg	59.8	±	10.1	62.7	±	10.7	0.295
BMI	kg/cm^2^	22.1	±	3.0	22.7	±	2.6	0.400
Systolic blood pressure	mmHg	119.1	±	13.7	116.7	±	11.3	0.482
Diastolic blood pressure	mmHg	76.4	±	7.8	74.5	±	11.1	0.459
Pulse rate	beat/min	71.3	±	10.7	70.9	±	10.3	0.872
PSQI-J global score		7.7	±	1.4	7.6	±	1.9	0.807
Test sample consumption	%	100.0	±	0.0	100.0	±	0.0	-

Values are presented as means ± SD. No significant differences were observed between groups (Fisher’s exact test or *t*-test).

## Data Availability

The data used and/or analyzed in this study are available from the corresponding author upon request.

## Supplementary Materials

The following supporting information can be downloaded at: https://www.mdpi.com/article/10.3390/nu16111702/s1, Table S1. Exclusion criteria. Table S2. Summary of EEG-derived sleep parameters. Table S3. Summary of the OSA-MA analysis on sleep quality. Table S4. Results of two-way ANOVA analysis for the effects of Test food and Sex on the measured parameters. Table S5. Results of two-way ANOVA analysis for the effects of Test food and Age on the measured parameters. Table S6. Assessment of sleepiness, physical condition, and biological indicators using questionnaires. Table S7. Summary of EEG-derived sleep parameters in the subgroup analysis. Table S8. Summary of the OSA-MA analysis on sleep quality in the subgroup analysis. Table S9. Assessment of sleepiness, physical condition, and biological indicators using questionnaires in the subgroup analysis. Table S10. Summary of safety assessment.
