# Supplementary material for: Bifidobacterium adolescentis SBT2786 Improves Sleep Quality in Japanese Adults with Relatively High Levels of Stress: A Randomized, Double-Blind, Placebo-Controlled Study
Source: Nutrients. 2024 May 30;16(11):1702. doi: 10.3390/nu16111702 (PMC11174696; doi:10.3390/nu16111702)
Supplement: Supplementary file 1 [file nutrients-16-01702-s001.zip › nutrients-2963631-supplementary.pdf]

Table S1. Exclusion criteria.

No.	Exclusion criteria
1	Participants regularly consuming foods for specified health uses, foods with function claims, or health foods that could potentially interfere with the study, and inability to discontinue their consumption after giving consent.
2	Participants using of medications that could interfere with the study and inability to limit their use during the study period.
3	Participants diagnosed with of sleep disorders such as insomnia, sleep apnea, restless legs syndrome, overactive bladder, or reflux esophagitis.
4	Participants with sleep affected by severe rhinitis or hay fever; usage of a pacemaker, inability to abstain from alcohol during the examination period, smokers, individuals with a history or current diagnosis of depression or other psychiatric disorders, abnormal glucose metabolism based on pre-test results.
5	Participants with history or current diagnosis of serious diseases of the heart, liver, kidneys, digestive organs, etc..
6	Participants employed in day and night shifts, late-night shifts, or jobs with large workload fluctuations and extremely irregular life rhythms.
7	Participants planning life events during the study period that would significantly change their lifestyle.
8	Participants planning for long-term business trips, travel, or events requiring overnight stays during the EEG measurement period, preventing EEG measurements.
9	Participants with frequent urination at night (more than twice a day), lack of a solitary sleeping environment;
10	Participants living in an environment that prevents solitary sleep (e.g., living with a person in need of care, an infant, a pet, etc.).
11	Participants diagnosed of dry mouth, easy bleeding from the oral cavity.
12	Participants who are pregnancy, lactation, or plans to become pregnant during the study.
13	Participants with drug or food allergies, current participation in another clinical trial or intention to participate in another clinical trial within 4 weeks after study completion.
14	Participants who donated component blood or 200 mL of whole blood one month prior to the study.
15	Male participants who donated 400 mL of whole blood 3 months prior to study entry.
16	Female participants who donated 400 mL of whole blood 4 weeks prior to study entry.
17	Male participants whose total blood collection in the 12 months prior to study entry plus the total planned blood collection for the study exceeding 1200 mL.
18	Female participants whose total blood collection in the 12 months prior to study entry plus the total planned blood collection for the study exceeding 800 mL.
19	Participants who are prone to skin irritation at the electrode site (head and neck).
20	Participants deemed by the investigator or sub-investigator to be ineligible for this study.

Table S2. Summary of EEG-derived sleep parameters.

Parameters	unit	time	Placebo			SBT2786			*p*-value (*t*-test)	*p*-value (ANCOVA)
Total sleep time (TST)	min	Week0	343.53	±	42.10	343.10	±	45.34	0.956	0.049*
		Week4	335.19	±	44.08	347.40	±	46.39	0.132	
Sleep period time (SPT)	min	Week0	377.58	±	48.41	376.23	±	49.47	0.877	0.011*
		Week4	366.80	±	49.31	383.13	±	49.68	0.066	
Sleep efficiency(TST/TIB)	%	Week0	84.57	±	5.37	84.33	±	5.00	0.797	0.617
		Week4	83.08	±	6.87	83.42	±	5.29	0.756	
Sleep latency (SL)	min	Week0	24.72	±	20.86	24.70	±	17.67	0.995	0.690
		Week4	30.04	±	22.75	28.80	±	20.36	0.748	
Total N3 stage	min	Week0	18.22	±	19.11	18.11	±	20.86	0.976	0.443
		Week4	18.45	±	20.09	17.27	±	19.68	0.740	
Total REM sleep time	min	Week0	69.61	±	16.76	70.68	±	18.27	0.732	0.034*
		Week4	63.88	±	14.01	69.73	±	19.34	0.056	
Total wakefulness time	min	Week0	34.06	±	15.12	33.13	±	13.81	0.722	0.039*
		Week4	31.61	±	14.26	35.73	±	14.87	0.115	
Delta power in SPT	μV^2/min	Week0	1180.30	±	628.66	1150.67	±	603.63	0.788	0.214
		Week4	1164.62	±	566.34	1065.83	±	518.03	0.310	
Wake time in last 2 hours of sleep	min	Week0	16.78	±	9.28	17.11	±	11.30	0.860	0.408
		Week4	17.13	±	13.58	15.71	±	10.29	0.509	

Values are presented as means ± SD. *Significant differences between the SBT2786 and placebo groups ($p < 0.05$) using *t*-test or analysis of covariance (ANCOVA), with each initial value as a covariate. TIB, time in bed; REM, rapid eye movement; N3, non-REM sleep stage 3.

Table S3. Summary of the OSA-MA analysis on sleep quality.

Parameters	time	Placebo	SBT2786	*p*-value (*t*-test)	*p*-value (ANCOVA)
Sleepiness on rising	week0	13.96 ± 5.12	14.73 ± 4.09	0.353	0.552
	week4	15.33 ± 5.11	16.25 ± 5.05	0.313	
Initiation and maintenance of sleep	week0	12.73 ± 3.79	13.31 ± 3.18	0.361	0.980
	week4	14.50 ± 4.34	14.66 ± 3.60	0.818	
Frequent dreaming	week0	21.72 ± 6.91	21.67 ± 6.84	0.966	0.756
	week4	21.14 ± 7.62	21.41 ± 7.03	0.837	
Refreshed on rising	week0	14.47 ± 4.15	15.23 ± 4.20	0.311	0.276
	week4	15.35 ± 5.02	16.54 ± 4.08	0.148	
Sleep length	week0	15.64 ± 4.36	15.54 ± 4.99	0.904	0.929
	week4	15.99 ± 4.69	16.02 ± 4.73	0.973	

Values are presented as means ± SD. No significant differences were observed between the groups using *t*-test or ANCOVA, with each initial value as a covariate.

Table S4. Results of two-way ANOVA analysis for the effects of Test food and Sex on the measured parameters.

Source of variation		*df*	Mean sq	*F*-value	*p*-value
EEG-derived sleep parameters					
Total sleep time (TST)					
	Test food	1	4694	2.31	0.131
	Sex	1	4760	2.34	0.129
	Interaction	1	428	0.21	0.647
Sleep period time (SPT)					
	Test food	1	8398	3.40	0.068
	Sex	1	2209	0.90	0.346
	Interaction	1	252	0.10	0.750
Sleep efficiency(TST/TIB)					
	Test food	1	3.5	0.09	0.761
	Sex	1	81.02	2.15	0.145
	Interaction	1	28.66	0.76	0.385
Sleep latency (SL)					
	Test food	1	48.7	0.10	0.748
	Sex	1	5.6	0.01	0.913
	Interaction	1	716.4	1.53	0.219
Total N3 stage					
	Test food	1	44	0.12	0.732
	Sex	1	3769	10.18	0.002*
	Interaction	1	140	0.38	0.540
Total REM sleep time					
	Test food	1	1072.7	3.79	0.054
	Sex	1	481.3	1.70	0.195

	Interaction	1	0	0.00	0.995
Total wakefulness time					
	Test food	1	536.7	2.54	0.114
	Sex	1	480.5	2.27	0.134
	Interaction	1	23.3	0.11	0.740
Delta power in SPT					
	Test food	1	307105	1.13	0.291
	Sex	1	2811402	10.30	0.002*
	Interaction	1	508014	1.86	0.175
Wake time in last 2 hours of sleep					
	Test food	1	63.1	0.46	0.498
	Sex	1	1445.2	10.57	0.001*
	Interaction	1	27.7	0.20	0.653
OSA-MA score					
Sleepiness on rising					
	Test food	1	26.52	1.06	0.304
	Sex	1	6.07	0.24	0.623
	Interaction	1	153.45	6.16	0.015*
Initiation and maintenance of sleep					
	Test food	1	0.845	0.05	0.820
	Sex	1	1.632	0.10	0.752
	Interaction	1	0.013	0.00	0.978
Frequent dreaming					
	Test food	1	2.3	0.04	0.838
	Sex	1	0.02	0.00	0.987
	Interaction	1	23.33	0.43	0.515
Refreshed on rising					
	Test food	1	44.57	2.13	0.147
	Sex	1	25.84	1.23	0.269
	Interaction	1	28.63	1.37	0.245
Sleep length					
	Test food	1	0.026	0.00	0.973
	Sex	1	7.729	0.35	0.557
	Interaction	1	14.754	0.66	0.418

*Significant ($p < 0.05$) using Two-way ANOVA

Table S5. Results of two-way ANOVA analysis for the effects of Test food and Age on the measured parameters.

Source of variation		*df*	Mean sq	*F*-value	*p*-value
EEG-derived sleep parameters					
Total sleep time (TST)					
	Test food	1	4694	2.29	0.133

	Age	2	1534	0.75	0.476
	Interaction	2	2015	0.98	0.378
Sleep period time (SPT)					
	Test food	1	8398	3.41	0.067
	Age	2	731	0.30	0.744
	Interaction	2	3356	1.36	0.260
Sleep efficiency(TST/TIB)					
	Test food	1	3.5	0.09	0.763
	Age	2	35.54	0.93	0.399
	Interaction	2	13.64	0.36	0.701
Sleep latency (SL)					
	Test food	1	48.7	0.10	0.750
	Age	2	8.9	0.02	0.982
	Interaction	2	308.6	0.65	0.526
Total N3 stage					
	Test food	1	43.7	0.12	0.731
	Age	2	1931.3	5.23	0.007*
	Interaction	2	462.7	1.25	0.289
Total REM sleep time					
	Test food	1	1072.7	3.79	0.054
	Age	2	66.1	0.23	0.792
	Interaction	2	468.3	1.66	0.195
Total wakefulness time					
	Test food	1	536.7	2.60	0.110
	Age	2	551.5	2.67	0.074*
	Interaction	2	177.3	0.86	0.427
Delta power in SPT					
	Test food	1	307105	1.08	0.301
	Age	2	1130451	3.98	0.021
	Interaction	2	135478	0.48	0.622
Wake time in last 2 hours of sleep					
	Test food	1	63.06	0.43	0.515
	Age	2	60.27	0.41	0.666
	Interaction	2	135.15	0.91	0.404
OSA-MA score					
Sleepiness on rising					
	Test food	1	26.524	1.01	0.316
	Age	2	26.089	1.00	0.372
	Interaction	2	5.122	0.20	0.822
Initiation and maintenance of sleep					
	Test food	1	0.845	0.05	0.818
	Age	2	24.88	1.56	0.214

	Interaction	2	11.277	0.71	0.495
Frequent dreaming					
	Test food	1	2.3	0.04	0.838
	Age	2	66.87	1.23	0.297
	Interaction	2	1.45	0.03	0.974
Refreshed on rising					
	Test food	1	44.57	2.08	0.152
	Age	2	18.04	0.84	0.434
	Interaction	2	1.4	0.07	0.937
Sleep length					
	Test food	1	0.03	0.00	0.973
	Age	2	1.86	0.08	0.920
	Interaction	2	41.99	1.90	0.155

Age : divided into age strata of 30s, 40s, and 50s

*Significant (p < 0.05) using Two-way ANOVA

Table S6. Assessment of sleepiness, physical condition, and biological indicators using questionnaires.

	unit	time	Placebo	SBT2786	*p*-value (*t*-test)	*p*-value (ANCOVA)
PSQI-J global score		week0	7.35 ± 2.03	7.18 ± 1.85	0.618	0.550
		week4	5.94 ± 2.17	5.66 ± 1.97	0.446	
ESS		week0	10.55 ± 5.14	10.52 ± 4.57	0.973	0.065
		week4	9.62 ± 5.03	8.53 ± 4.59	0.207	
VAS (sleep)		week0	44.00 ± 15.55	45.36 ± 13.52	0.602	0.093
		week4	47.90 ± 14.97	52.48 ± 14.10	0.080	
POMS2 TMD score		week0	50.32 ± 8.50	50.34 ± 7.92	0.988	0.041*
		week4	48.88 ± 8.16	46.84 ± 7.43	0.145	
VAS (fatigue)		week0	54.40 ± 15.19	53.92 ± 14.83	0.858	0.543
		week4	52.65 ± 14.28	51.20 ± 10.95	0.522	
Salivary amylase	U/mL	week0	89.10 ± 37.39	92.80 ± 41.77	0.601	0.764
		week4	109.34 ± 51.11	115.13 ± 58.52	0.555	
Blood growth hormone	ng/mL	week0	1.06 ± 1.30	1.55 ± 2.35	0.156	0.642
		week4	0.89 ± 1.24	0.91 ± 1.12	0.902	

Values are presented as means ± SD. *Significant differences between the SBT2786 and placebo groups (p < 0.05) using ANCOVA, with each initial value as a covariate.

Table S7. Summary of EEG-derived sleep parameters in the subgroup analysis.

Parameters	unit	time	Placebo			SBT2786			*p*-value (*t*-test)	*p*-value (ANCOVA)
Total sleep time (TST)	min	Week0	344.89	±	37.41	356.07	±	35.38	0.261	0.061
		Week4	330.16	±	44.38	358.25	±	47.82	0.028*	
Sleep period time (SPT)	min	Week0	380.47	±	42.64	387.86	±	39.56	0.510	0.040*
		Week4	362.81	±	48.77	391.37	±	49.28	0.036*	
Sleep efficiency(TST/TIB)	%	Week0	84.58	±	4.85	85.28	±	5.16	0.607	0.105
		Week4	81.28	±	7.98	84.70	±	6.64	0.092	
Sleep latency (SL)	min	Week0	22.56	±	15.88	20.91	±	11.66	0.666	0.469
		Week4	33.51	±	26.33	27.91	±	22.18	0.401	
Total N3 stage	min	Week0	16.16	±	19.68	24.36	±	23.40	0.164	0.992
		Week4	15.71	±	20.27	23.34	±	22.23	0.189	
Total REM sleep time	min	Week0	70.49	±	19.43	70.53	±	13.51	0.993	0.093
		Week4	64.95	±	12.59	71.68	±	19.24	0.137	
Total wakefulness time	min	Week0	35.58	±	16.14	31.79	±	13.75	0.355	0.483
		Week4	32.65	±	15.37	33.12	±	16.01	0.913	
Delta power in SPT	$\mu V^2/min$	Week0	1067.83	±	515.30	1345.75	±	694.72	0.096	0.858
		Week4	1055.03	±	563.10	1273.86	±	590.64	0.166	
Wake time in last 2 hours of sleep	min	Week0	18.16	±	10.51	18.63	±	14.00	0.886	0.050*
		Week4	20.66	±	18.01	13.98	±	10.35	0.094	

Values are presented as means ± SD. *Significant differences between the SBT2786 and placebo groups ($p < 0.05$) using *t*-test or ANCOVA, with each initial value as a covariate. TIB, time in bed; REM, rapid eye movement; N3, non-REM sleep stage 3.

Table S8. Summary of the OSA-MA analysis on sleep quality in the subgroup analysis.

Parameters	time	Placebo			SBT2786			*p*-value (*t*-test)	*p*-value (ANCOVA)
Sleepiness on rising	week0	13.99	±	5.64	15.24	±	4.81	0.385	0.038*
	week4	14.23	±	5.18	17.14	±	4.95	0.038*	
Initiation and maintenance of sleep	week0	12.75	±	4.03	14.13	±	3.21	0.169	0.383
	week4	13.69	±	4.35	14.79	±	3.07	0.288	
Frequent dreaming	week0	21.47	±	7.18	20.94	±	6.99	0.784	0.171
	week4	19.52	±	8.10	21.58	±	6.65	0.312	
Refreshed on rising	week0	13.91	±	3.99	16.10	±	5.23	0.085	0.028*
	week4	14.16	±	4.31	17.44	±	3.94	0.005*	
Sleep length	week0	15.45	±	4.63	15.42	±	5.48	0.978	0.231
	week4	14.56	±	4.20	15.81	±	4.78	0.306	

Values are presented as means ± SD. *Significant differences between the SBT2786 and placebo groups ($p < 0.05$) using *t*-test or ANCOVA, with each initial value as a covariate.

Table S9. Assessment of sleepiness, physical condition, and biological indicators using questionnaires in the subgroup analysis.

	unit	time	Placebo			SBT2786			*p*-value (*t*-test)	*p*-value (ANCOVA)
PSQI-J global score		week0	7.55	±	1.94	7.12	±	1.90	0.404	0.032*
		week4	6.55	±	1.82	5.27	±	2.16	0.021*	
ESS		week0	9.94	±	5.55	10.44	±	4.73	0.723	0.011*
		week4	9.67	±	5.58	7.83	±	4.93	0.203	
VAS (sleep)		week0	46.68	±	15.01	48.54	±	15.63	0.654	0.039*
		week4	47.14	±	16.84	56.02	±	13.51	0.037*	
POMS2 TMD score		week0	50.21	±	9.06	50.46	±	8.22	0.914	0.039*
		week4	48.69	±	8.30	45.96	±	7.14	0.199	
VAS (fatigue)		week0	53.10	±	17.09	52.31	±	14.38	0.853	0.692
		week4	54.06	±	16.92	52.41	±	9.52	0.655	
Salivary amylase	U/mL	week0	122.93	±	27.83	128.76	±	38.25	0.518	0.851
		week4	141.71	±	51.86	150.57	±	68.44	0.588	
Blood growth hormone	ng/mL	week0	1.26	±	1.30	1.26	±	1.75	0.985	0.422
		week4	0.91	±	1.00	0.71	±	0.82	0.432	

Values are presented as means ± SD. *Significant differences between the SBT2786 and placebo groups ($p < 0.05$) using *t*-test or ANCOVA, with each initial value as a covariate.

Table S10. Summary of safety assessment.

			Placebo			SBT2786			*p*-value (*t*-test)
Body weight	kg	Week0	61.38	±	10.21	61.35	±	11.42	0.988
		Week4	61.31	±	10.34	61.08	±	11.28	0.901
BMI	kg/m^2	Week0	22.65	±	2.87	22.28	±	2.75	0.444
		Week4	22.62	±	2.90	22.19	±	2.72	0.367
systolic blood pressure	mmHg	Week0	113.47	±	13.40	115.20	±	13.86	0.454
		Week4	111.89	±	14.73	112.79	±	13.45	0.706
diastolic blood pressure	mmHg	Week0	71.77	±	9.39	71.83	±	10.32	0.973
		Week4	69.53	±	10.01	70.41	±	11.18	0.622
puls rate	bpm	Week0	72.47	±	10.09	76.91	±	10.72	0.013*
		Week4	71.66	±	9.48	75.37	±	10.45	0.029*
WBC	/μL	Week0	5454.00	±	1529.43	5681.86	±	1486.49	0.373
		Week4	5188.00	±	1452.40	5314.57	±	1368.81	0.597
RBC	$\times 10^4$/μL	Week0	474.50	±	42.14	469.46	±	46.87	0.504
		Week4	457.44	±	46.15	454.24	±	45.51	0.680
Hb	g/dL	Week0	14.23	±	1.37	14.06	±	1.41	0.455
		Week4	13.72	±	1.48	13.58	±	1.34	0.551
Ht	%	Week0	43.99	±	3.56	43.43	±	3.73	0.359
		Week4	42.64	±	4.03	42.17	±	4.05	0.489
Plt	$\times 10^4$/μL	Week0	26.95	±	5.23	27.54	±	5.43	0.518
		Week4	26.41	±	4.69	26.33	±	5.15	0.926
TP	g/dL	Week0	7.26	±	0.34	7.26	±	0.32	0.939
		Week4	7.00	±	0.34	7.02	±	0.31	0.738
Alb	g/dL	Week0	4.49	±	0.24	4.55	±	0.25	0.141
		Week4	4.31	±	0.25	4.40	±	0.24	0.026*
T-Bil	mg/dL	Week0	0.71	±	0.22	0.69	±	0.26	0.779
		Week4	0.68	±	0.22	0.63	±	0.24	0.295
ALP/IFCC	U/L	Week0	67.10	±	17.79	65.41	±	19.03	0.589
		Week4	61.76	±	15.06	60.41	±	16.79	0.619
LD/IFCC	U/L	Week0	163.39	±	21.11	162.97	±	23.10	0.912
		Week4	168.00	±	22.49	167.91	±	23.13	0.982
AST	U/L	Week0	21.73	±	6.07	21.69	±	4.25	0.961
		Week4	20.63	±	5.26	20.31	±	4.40	0.702
ALT	U/L	Week0	20.54	±	11.35	20.37	±	10.35	0.926
		Week4	18.46	±	10.83	16.67	±	6.50	0.239
γ-GT	U/L	Week0	25.87	±	16.16	25.24	±	17.04	0.823
		Week4	25.70	±	24.99	22.46	±	13.02	0.338
CK	U/L	Week0	105.69	±	49.86	110.20	±	54.84	0.611
		Week4	113.77	±	64.12	125.81	±	87.28	0.354

T-Cho	mg/dL	Week0	208.23	±	32.72	207.93	±	30.33	0.955
		Week4	197.06	±	29.67	198.21	±	28.59	0.815
TG	mg/dL	Week0	78.56	±	35.68	80.00	±	43.39	0.830
		Week4	80.67	±	50.27	79.61	±	48.65	0.900
HDL-C	mg/dL	Week0	67.97	±	14.80	67.57	±	17.87	0.886
		Week4	63.70	±	13.46	64.56	±	16.90	0.740
LDL-C	mg/dL	Week0	120.37	±	27.00	119.80	±	25.82	0.898
		Week4	114.87	±	26.47	116.13	±	24.39	0.771
UN	mg/dL	Week0	12.78	±	3.14	12.63	±	3.33	0.783
		Week4	12.58	±	3.16	12.42	±	3.31	0.774
CRE	mg/dL	Week0	0.74	±	0.14	0.72	±	0.17	0.545
		Week4	0.75	±	0.13	0.76	±	0.15	0.629
UA	mg/dL	Week0	5.01	±	1.11	5.04	±	1.34	0.902
		Week4	4.97	±	1.13	5.13	±	1.33	0.469
Na	mEq/L	Week0	140.87	±	1.68	140.71	±	1.65	0.577
		Week4	139.57	±	1.42	139.13	±	1.87	0.117
K	mEq/L	Week0	4.47	±	0.34	4.53	±	0.34	0.312
		Week4	4.17	±	0.30	4.18	±	0.32	0.827
Cl	mEq/L	Week0	104.13	±	1.76	103.44	±	1.74	0.022*
		Week4	104.46	±	1.72	103.76	±	1.77	0.019*
Ca	mg/dL	Week0	9.45	±	0.30	9.44	±	0.29	0.818
		Week4	9.21	±	0.26	9.18	±	0.30	0.572
GLU	mg/dL	Week0	90.80	±	6.23	90.37	±	6.50	0.691
		Week4	89.17	±	6.31	90.16	±	7.94	0.417
HbA1c(NGSP)	%	Week0	5.37	±	0.25	5.40	±	0.27	0.499
		Week4	5.37	±	0.21	5.40	±	0.27	0.447

Values are means ± SD. * Significant differences between SBT2786 and placebo group ($p < 0.05$) by *t*-test
